# Monitoring southwest Greenland’s ice sheet melt with ambient seismic noise

**DOI:** 10.1126/sciadv.1501538

**Published:** 2016-05-06

**Authors:** Aurélien Mordret, T. Dylan Mikesell, Christopher Harig, Bradley P. Lipovsky, Germán A. Prieto

**Affiliations:** 1 Department of Earth, Atmospheric and Planetary Sciences, Massachusetts Institute of Technology, Cambridge, MA 02139–4307, USA.; 2 Department of Geosciences, Princeton University, Princeton, NJ 08544, USA.; 3 Department of Geophysics, Stanford University, Stanford, CA 94305–2004, USA.; 4 Department of Earth and Planetary Sciences, Harvard University, Cambridge, MA 02138, USA.

**Keywords:** East Antarctic Ice Sheet, Antarctica, sea ice, glaciers, Wilkes Land

## Abstract

The Greenland ice sheet presently accounts for ~70% of global ice sheet mass loss. Because this mass loss is associated with sea-level rise at a rate of 0.7 mm/year, the development of improved monitoring techniques to observe ongoing changes in ice sheet mass balance is of paramount concern. Spaceborne mass balance techniques are commonly used; however, they are inadequate for many purposes because of their low spatial and/or temporal resolution. We demonstrate that small variations in seismic wave speed in Earth’s crust, as measured with the correlation of seismic noise, may be used to infer seasonal ice sheet mass balance. Seasonal loading and unloading of glacial mass induces strain in the crust, and these strains then result in seismic velocity changes due to poroelastic processes. Our method provides a new and independent way of monitoring (in near real time) ice sheet mass balance, yielding new constraints on ice sheet evolution and its contribution to global sea-level changes. An increased number of seismic stations in the vicinity of ice sheets will enhance our ability to create detailed space-time records of ice mass variations.

## Teaser

Researchers monitor southwest Greenland’s ice sheet mass changes by measuring seismic velocity variations in Greenland’s crust.

## INTRODUCTION

**Fig. 1:**
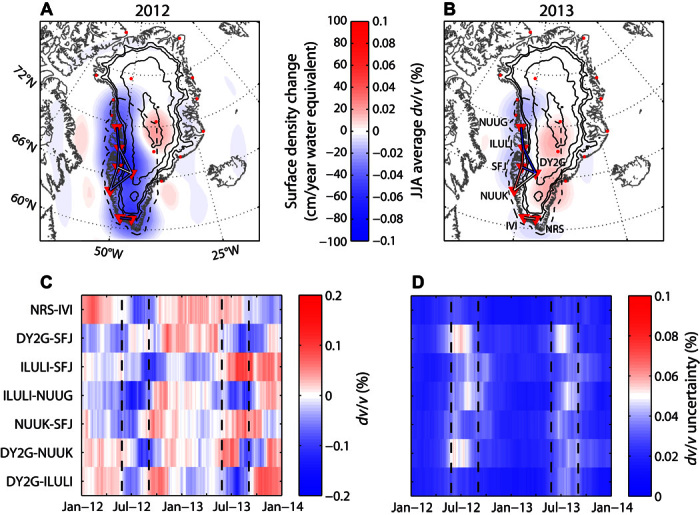
Ice mass balance and measured velocity variations. (**A**) Map of the ice mass changes over southwest Greenland in 2012. The stations used in this study are indicated by inverted triangles; the other Greenland stations of the Greenland Ice Sheet Monitoring Network (GLISN) are indicated by red dots. The colored lines between the stations show the *dv*/*v* averaged over the summer months June, July, and August (JJA). The black contours show the ice thickness at a 1000-m increment; the dashed black curve shows the contour used to integrate the ice mass changes from GRACE data. (**B**) Same as (A) for 2013. (**C**) Time series of the relative velocity variations. (**D**) Time series of the relative velocity variation uncertainties. The summer months are between dashed vertical lines.

Monitoring large-scale natural phenomena that occur in remote environments, such as ice sheet mass balance, with high spatial and temporal resolution is very challenging. Although airborne and spaceborne techniques have constrained decadal trends in the Greenland ice sheet (GIS) mass balance (*1*–*3*), short-term–like seasonal fluctuations pose an ongoing challenge. Notable short-term fluctuations include the record-breaking 2012 melting event (*4*) (Fig. 1A) and the absence of noticeable melt in 2013 (*5*) (Fig. 1B). Such short-term fluctuations may be undersampled by airborne or spaceborne techniques and may therefore bias the long-term decadal trend estimation. The result of an obscured long-term mass balance trend may be severe: such a situation may result in bias in sea-level rise projections and potentially have a significant political and societal impact on vulnerable populations (*6*–*8*).

GIS mass balance is currently monitored at a variety of different temporal and spatial scales. Gravity Recovery and Climate Experiment (GRACE) data have a monthly resolution in time and an approximately 300-km resolution over the entire GIS (*9*). Airborne and satellite radar and laser altimetry have much higher spatial resolution but lack the short-term time resolution of GRACE as they provide at best two to three data acquisitions per year. Global Positioning System (GPS) measurement of ice velocity or crustal uplifting provides a spatially narrow point measurement of ice or ground motion; it is low-cost, is accurate, and has dense, subdaily sampling in time. Each of these methods requires certain assumptions to infer ice mass from observed quantities. GRACE, for example, must be corrected for long-term crustal deformation (*3*, *10*–*12*). Altimetric methods interpret elevation data as mass change by making assumptions on the density profile of the snow and ice or the compaction behavior of firn. These assumptions remain strongly debated (*13*).

We propose a new technique to measure ice sheet mass changes using Earth’s natural seismic field. This seismic-based approach may be used for continuous, potentially real-time monitoring of the seasonal ice mass variations of the GIS. We use the correlation of Earth’s ambient seismic noise, a technique originally developed to monitor active volcanoes (*14*–*17*) and active fault zones (*18*–*20*). With this technique, we measure seismic wave velocity variations in the Greenland crust due to ice sheet loading.

## RESULTS

### Velocity variation observations

We compute daily seismic noise correlation functions. On the basis of extensive theoretical and experimental work, we interpret the seismic noise correlation function between two stations as a proxy of Green’s function between these two stations (*21*–*24*). In real Earth data sets, where the seismic noise is dominated by surface waves, the surface wave part of Green’s function is the most easily retrieved, provided that the noise sources are homogeneously distributed around the stations. Each daily correlation function is then compared to a reference correlation function to assess relative seismic velocity changes (*dv*/*v*). In this monitoring application, a weaker temporal stability of the noise sources is sufficient for the method to be successful (*25*–*27*). The comparison is made in the coda part of the correlation function (the latter part of the signal composed of singly and multiply scattered seismic waves), which samples Earth over a much longer time than do ballistic waves. Coda waves are therefore more sensitive to small changes in the medium (*28*).

We analyze 2 years (2012–2013) of vertical-component continuous 1-Hz seismic data recorded in Greenland by seven stations in the GLISN network (*29*): NRS, IVI, NUUK, SFJ, DY2G, ILULI, and NUUG (Fig. 1B). These stations are located on the western side of Greenland, and we analyze only the station pairs separated by less than 400 km and with a signal-to-noise ratio (SNR) higher than 30 in the considered frequency band. Pairs with larger interstation distances did not have a SNR that was sufficiently high to provide accurate seismic velocity variation measurements (fig. S1). Given the peculiar characteristic of the seismic noise around Greenland (*30*), we analyze the seismic data in the frequency band 0.1 to 0.3 Hz, where the SNR of the correlations is the highest (fig. S1) and where the correlations are least biased by seasonal variations of the seismic noise (see “Seismic data analysis and processing” section in Materials and Methods and figs. S2 and S3). Moreover, in this frequency range, the seismic waves mostly sample the Greenland crust between depths of 3 and 10 km. After analysis of the different parameters involved in the *dv*/*v* measurements (see “*dv*/*v* measurement tests” section in Materials and Methods and associated figures), we chose to measure *dv*/*v* with the “stretching” technique (*14*, *25*) using a 300-s-long window starting at 1.3 × *t*_0_, where *t*_0_ is the direct Rayleigh wave arrival time. Only on the most energetic side of each correlation did we measure in the coda, and the daily correlations were first averaged over a 90-day moving window to stabilize the results and suppress the influence of transient perturbations such as tectonic (*19*, *20*) or glacial (*31*) earthquakes. The raw time series of the *dv*/*v* variations for each of the seven pairs of stations analyzed are shown in Fig. 1C.

We observe a clear, coherent decrease of seismic velocity during the summer months (Fig. 1, A to C). This decrease is less systematic in 2013 (Fig. 1, B and C). A denser seismic network would be necessary to assess whether this latter observation is due to actual regional differences in ice sheet melting or to errors in the velocity variation estimation process. For example, the strong decrease observed in 2013 for station pairs ILULI-NUUG and ILULI-DY2G could be due to the strong melt of the Jakobshavn glacier, which was heavily melting in 2013 although the rest of the ice sheet was not (*32*). ILULI station is located only a few kilometers away. The noise source variations during summer months introduce higher uncertainties (Fig. 1D); however, they do not interfere with our interpretations (see “Seismic data analysis and processing” section in Materials and Methods).

**Fig. 2:**
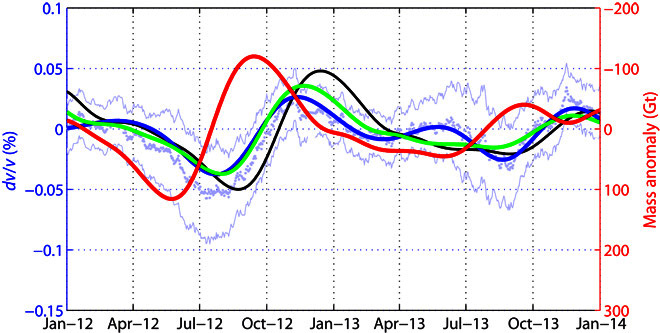
Velocity variation modeling. The thick red curve is the ice mass variation corrected from its quadratic trend and filtered in the 4- to 17-month period band. The green curve is the poroelastic modeling of *dv*/*v* based on the red curve. The black curve is the result of the viscoelastic modeling. The blue curve is the *dv*/*v* measurements averaged over all station pairs and filtered in the 4- to 17-month period, whereas the pale blue dots and the thin pale blue curves are the raw average *dv*/*v* measurements and the corresponding average uncertainties, respectively.

Given the sparsity of our network, we do not have the spatial resolution to interpret individual station pair measurements. Instead, we average *dv*/*v* over the seven pairs of stations and interpret a single, spatially averaged velocity change time series. This time series is assumed to be representative of the entire southwest Greenland region. We observe a velocity decrease (−0.05%) during the summer of 2012 and a smaller decrease (−0.025%) during the summer of 2013 (Fig. 2). We compare the raw, spatially averaged velocity variation time series to GRACE measurements (Fig. 2), and we find that seismic velocities lag behind changes in ice sheet mass inferred from GRACE measurements by ~2 to 3 months. For an annually periodic loading cycle, this corresponds to a phase lag of ~90°. We next explore two mechanical models to explain these observations.

### Mechanical origin of the observed velocity variation

The Greenlandic crust responds to seasonal loading and unloading of the annual snow pack by subsiding and uplifting. This forcing strains the crust and the underlying mantle. Strain may be accommodated by a variety of processes, including poroelastic and viscoelastic deformation [for example, pore pressure variations, opening and closing of cracks, and/or viscous flow in the mantle (*33*)]. However strain is accommodated, it translates into perturbations in the effective elastic moduli of the bedrock (*34*–*36*). We consider two end-member models of the crustal response to seasonal loading. In the first model, the crust-mantle system responds as a viscous medium, and in the second model, the crust responds as a poroelastic medium.

### Poroelastic model

We consider a nonlinear poroelastic model (*34*) that relates pore pressure variation to *dv*/*v*. We assume that the pore pressure variations *P*_p_(*t*) at the base of the glacier are approximately equal to glaciostatic pressure variations *P*_g_(*t*)

$$P_{p}(t)\approx P_{g}(t)=\frac{M_{i}(t)g}{S_{i}}$$ (1)

where *M*_*i*_(*t*) is the detrended ice mass variations measured by GRACE over southwest Greenland, *S*_*i*_ is the area of the GIS from which we integrated the ice mass (Fig. 1), and *g* is the gravitational acceleration. Here, we use the detrended version of the mass variation curve because our 2-year study period is too short to reliably extract long-term trend information from the seismic noise. Ice mass change, corrected from its quadratic trend and filtered between 4 and 17 months, is shown in Fig. 2.

*P*_p_(*t*) is then input into a modified version of the poroelastic model derived by Tsai (*34*), which includes a thin, incompetent layer at the surface with hydraulic properties different from the bedrock. On the basis of glaciology literature (*37*, *38*), we give this layer the hydraulic properties of glacial till, which can exist between the glacier and the bedrock. We obtain the relative velocity variation *dv*/*v*(*t*) as

$$dv/v(t)=A(t)e^{-kz}[\frac{1-2\nu }{E}((1-kz)+\frac{\lambda +3\mu +m}{\mu }(1-2\nu )\;\text{sin}\;kx)]$$ (2)

where *A*(*t*) is given by

$$A(t)=\frac{1+\nu }{1-\nu }k\alpha P_{p}(t-\Delta t)\sqrt{\frac{K_{c}}{\omega }}e^{\frac{\pi }{4}-\omega \Delta t}$$ (3)

and the time delay Δ*t* between the pore pressure at the surface and the velocity variation is given by

$$\Delta t=\frac{z_{t}}{\sqrt{2\omega K_{t}}}+\frac{\overset{-1}{\text{cot}}(\frac{K_{c}k^{2}}{\omega })}{2\omega }$$ (4)

The first term in Eq. 4 is the delay due to the till layer, and the second term is the delay due to pore pressure diffusion in the bedrock. In Eq. 2, the parameters are as follows: *k* is the wave number of the surface pressure field; *z* is the depth sampled by the seismic wave; $\nu =\frac{0.5-{\left(Vs/Vp\right)}^{2}}{1-{\left(Vs/Vp\right)}^{2}}$, is Poisson’s ratio of the upper crust, with *Vp* and *Vs* being the *P*- and *S*-wave velocities in the upper crust, respectively; $E=\frac{\rho_{c}Vp^{2}(1+\nu )(1-2\nu )}{1-\nu }$ is Young’s modulus, with ρ_c_ being the density of the upper crust; $\lambda =\frac{E\nu }{\left(1+\nu )(1-2\nu \right)}$ and $\mu =\frac{E}{2(1+\nu )}$ are Lamé’s parameters; *m* is the second Murnaghan third-order elastic constant; and *x* is the horizontal position of the seismometer taken as a representative distance from the melting ice sheet giving the largest *dv*/*v* signal. In Eq. 3, α is Biot’s coefficient, *K*_c_ is the hydraulic diffusivity of the crust, and ω is the angular frequency of the main period of the signal. In Eq. 4, *z*_t_ is the thickness of the till layer and *K*_t_ is the hydraulic diffusivity of the till. The numerical value of each parameter is given in Table 1, along with the references where they can be found. As already stated by Tsai (*34*), the modeled *dv*/*v* requires a Murnaghan constant value outside the realistic range to fit the data. However, this parameter is poorly constrained in the literature; therefore, we fit the predicted *dv*/*v* to the measured *dv*/*v* by adjusting the value of the Murnaghan coefficient *m* and the thickness of the till layer through a grid search. The results shown in fig. S5 give *m*/μ = −94.4 × 10^4^ and 2.85 m of glacial till, with μ = 29.4 GPa, the shear modulus of the Greenland crust. The best-fit model using these values is shown in Fig. 2 and correlates with the *dv*/*v* data filtered in the 4- to 17-month period at 91%.

**Table 1:** Parameters used in the *dv*/*v* modeling.

**Parameter**	**Symbol**	**Value**	**Reference**
Glaciostatic pressure	*P*_g_	1600 Pa	From data
Ice area	*S*_*i*_	6.5 × 10^11^ m^2^	From data
Gravitational acceleration	*g*	9.81 m/s^2^	
Pressure field wave number	*k*	2π/(60 km)	Jiang *et al*. (*11*)
Depth of investigation	*z*	5 km	From data
*S*-wave velocity	*Vs*	3300 m/s	Kumar *et al*. (*56*)
*Vp*/*Vs* ratio	*Vp*/*Vs*	1.8	Kumar *et al*. (*56*)
Upper-crust density	ρ_c_	2700 kg/m^3^	Schmidt-Aursch and Jokat (*57*)
*P*-wave velocity	*Vp* = *Vs*(*Vp*/*Vs*)	5940 m/s	
Poisson’s ratio	ν	0.2768	
Young’s modulus	*E*	7.5 × 10^10^ Pa	
Mantle viscosity	η	10^21^ Pa⋅s	
Viscoelastic relaxation time	*T*	10^11^ s	
Lamé’s first parameter	λ	3.65 × 10^10^ Pa	
Shear modulus	μ	2.94 × 10^10^ Pa	
Murnaghan constant	*m*	−2.77 × 10^16^ Pa	From inversion
Distance from the ice	*x*	12.5 km	
Biot’s coefficient	α	0.7	Tsai (*34*)
Hydraulic diffusivity of the crust	*K*_c_	0.5 m^2^/s	Shapiro *et al*. (*58*)
Angular frequency	ω	2π/(365 days)	
Till layer thickness	*z*_t_	2.85 m	From inversion
Hydraulic diffusivity of till	*K*_t_	5 × 10^− 6^ m^2^/s	Iverson *et al*. (*38*)

Bevis *et al*. (*12*) showed that the atmospheric pressure variation component in Greenland was of the same order of magnitude as the ice mass pressure variations, and the atmospheric pressure variation was necessary to explain the annual fluctuations of crustal displacement observed by GPS. However, atmospheric mass variations have a much larger spatial wavelength (~1000 km) than the ice mass variations and are therefore negligible in our poroelastic modeling.

The theoretical *dv*/*v* time series is a negative, amplitude-scaled, and time-delayed version of the (detrended) pore pressure input derived from GRACE. It has to be noted that this poroelastic model does not actually satisfy the fully coupled governing equations of linear poroelasticity. Furthermore, the prescribed-pressure boundary condition is also an approximation that may require more detailed analysis in the future. However, given the uncertainty in the data, this first-order approach should be sufficient.

### Viscous rebound model

We consider a model where the observed phase lag between velocity perturbations and ice mass loading arises due to the viscous response of the crust-mantle system. We describe this system using the linear stress-strain relation $\sigma =\eta \dot{\epsilon }$ for stress σ, strain rate $\dot{\epsilon }$, and viscosity η. We note that a viscous response may be justified in the short-time limit of the more general Kelvin viscoelastic rheology. Such an approximation is justified because we expect the viscoelastic relaxation time (*T* ≡ η/*E* ≈ 10^11^ s (*39*), where *E* is Young’s modulus) to be much longer than the period of annual forcing.

This linear stress-strain relation predicts a 90° phase shift between an applied ice load and straining at depth. As described in the previous section, we observe a phase delay of several months between ice mass loading and seismic velocity changes at depth (Fig. 2). Within this viscous model, the phase lag is explained as the time required for viscous strains to accumulate in response to ice mass fluctuations.

We further quantify the predictions of this viscous model by calculating strain amplitudes at depth. We calculate the spatial pattern of stress changes due to the uniformly distributed glaciostatic pressure *P*_*g*_(*t*) at the bed of the ice sheet (fig. S4) (*40*). Stresses rapidly decrease away from the ice sheet, with a length scale proportional to the wavelength of pressure change ~50 km. We then calculate the volume-averaged stress change in this region sampled by our observations: the upper 5 km of Earth’s crust in a 50-km region centered at the ice sheet margin. The average stress change is found to be ≈ 0.5*P*_*g*_(*t*), giving a velocity-stress sensitivity of ~ 10^−7^ Pa^−1^ (velocity-strain sensitivity, 0.5% per microstrain), in agreement with values found in other studies (*41*–*43*).

The estimated viscoelastic seismic velocity variations are shown by the black curve in Fig. 2. The phase shift between the ice loading and the velocity variations is overestimated by ~20 days and gives a correlation of 77% with the *dv*/*v* data filtered in the 4- to 17-month period band.

## DISCUSSION

**Fig. 3:**
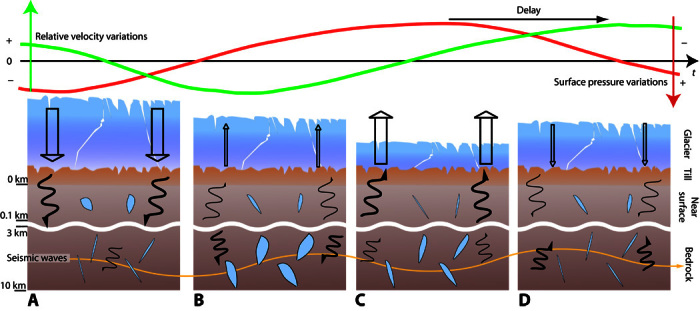
Pore pressure diffusion in the Greenland crust through a till layer. The straight arrow indicates surface pressure and the wavy arrow indicates pore pressure diffusion; the thickness of the arrows indicates the amplitude of the pressure change. (**A**) Maximum pressure at Earth’s surface due to snow accumulation. The pore pressure change at depth is delayed by the bedrock and the till layer. The change is felt in the near surface and then starts to be felt at depth. (**B**) The maximum pore pressure change has reached the depth sampled by the seismic waves, and the relative velocity variation is therefore minimum. Meanwhile, surface pressure decreases as a result of ice melting and atmospheric pressure variations. (**C**) The surface pressure change reaches its minimum, followed by the pore pressure in the near surface. At depth, the pore pressure continues to decrease, whereas the velocity continues to increase. (**D**) Surface pressure starts to increase again. At depth, pore pressure is minimum, and relative velocity variation is maximum. Sections are not to scale.

We developed two end-member models to explain the observed seismic velocity changes in Greenland. Because of the geological characteristics of the studied area (fluids and presence of till) and because of the better fit with our data, we favor the poroelastic model. However, it is probable that the observed velocity changes are a combination of both poroelastic and viscoelastic effects, and we need more data to definitively address this question. In the case of the poroelastic model, we interpret the observed velocity changes as the effect of a pore pressure wave diffusing in the Greenland crust and being modulated by the pressure variations of the ice changes at the surface (Fig. 3). The observed delay between the surface pressure and the velocity variations is mainly controlled by the hydraulic properties of the till layer and of the bedrock. Although the best model suggests a 2.85-m till layer, the *dv*/*v* uncertainties are too large to determine whether this parameter is necessary, given the poorly known Murnaghan constant. Using more data such as the records from the horizontal components of the seismic sensors could help reduce this uncertainty.

Moreover, it is possible that the seismic noise correlation technique may not be sensitive to the long-term decrease of ice mass. The reduction of the ice sheet mass induces a decrease in pore pressure at depth. However, this pore pressure decrease is limited by the complete closure of the cracks in the bedrock: even if the ice mass continues to decrease, the seismic velocity can no longer increase once all cracks and pores are sealed. Nevertheless, an increase of ice mass can always increase the pore pressure and reopen the pores, whatever the former pressure state of the crust, leading to a seismic velocity decrease. The seismic noise correlation technique may, therefore, be more sensitive to the relative mass variations between winter and summer than to the absolute ice mass changes.

Our analysis demonstrates that the seismic noise correlation technique can be used to continuously monitor the changes occurring in the GIS. Our successful modeling of the observed seismic velocity variations suggests that, knowing the long-term trend of ice mass loss variations, we can retrieve the GIS ice mass change locally from seismic data. The sparsity of our network does not allow us to interpret with certainty the spatial variations of seismic velocity from individual pairs of stations. However, we propose that, with a denser seismic network, one could produce accurate tomographic maps (*20*) of ice mass changes over the whole GIS. This method might also be used to infer the local thickness of a till layer, a poorly known parameter that strongly influences ice dynamics (*37*, *44*).

## MATERIALS AND METHODS

### Seismic data analysis and processing

The seismic noise in Greenland is highly variable, mostly due to the presence of sea ice in winter. Sergeant *et al*. (*30*) extensively studied the distribution of seismic noise sources around Greenland as a function of the seismic wave period. They showed that the influence of sea ice is not negligible for periods smaller than 4 s. In the presence of sea ice, short-period seismic noise is strongly attenuated, and it becomes more energetic as the sea ice disappears. However, this effect is weaker near the southern stations that we used in this study. This is clearly illustrated by comparing the spectrograms of station KULLO (74.5805°N, −57.2201°W; not used in this study) and station NRS (fig. S2). In winter, sea ice prevents wave interference (*45*) in Baffin Bay, where the shallow bathymetry enhances short-period seismic noise. The other short-period sources, south of Greenland, are too far away; this energy is attenuated before it reaches the northern stations. However, in summer, without sea ice, short-period seismic noise is easily excited in Baffin Bay and the short-period noise level reaches the level of the long-period noise (5 to 10 s). In general, the 3- to 10-s band contains the most energy. At periods longer than 10 s, the seismic noise again shows a strong seasonal trend, with a clear minimum in summer. Moreover, at these long periods, the Greenland crust should be weakly scattering; this would produce a short and weak coda in the correlations, leading to little velocity variation information.

We used the MSNoise Python package (*46*) to compute daily noise correlations between seven pairs of stations separated by less than 400 km (sorted by increasing interstation distance): NRS-IVI, DY2G-SFJ, ILULI-SFJ, ILULI-NUUG, NUUK-SFJ, DY2G-NUUK, and DY2G-ILULI. We downloaded the data from the Incorporated Research Institutions for Seismology (IRIS) facility and deconvolved the instrument response using obspyDMT software (*47*). The noise preprocessing is as follows: each daily trace is cut into 4-hour-long segments (*48*, *49*), and the segments are demeaned, detrended, and filtered between 0.01 and 0.4 Hz. Then, amplitudes larger than 3 standard deviations are normalized, and each segment is spectrally whitened between 0.01 and 0.4 Hz. The segments are finally correlated between the different pairs of stations, and the correlations are stacked to obtain daily cross-correlations. Figure S3 shows that seasonal variation in seismic noise does affect the coherency of the daily correlations, with a lower coherency in summer. Indeed, in the 3- to 10-s period band in summer, most of the noise sources are in the Southern Hemisphere. The new sources appearing where the sea ice melts near Greenland are higher frequencies and do not strongly contribute to the coherency of the correlations. However, the seasonal variations mostly influence the frequency domain amplitude spectra. Although we found small changes in the frequency spectrum, the main frequency peaks remain observable throughout the analyzed period (fig. S3, D and E). This indicates that noise preprocessing is efficient in mitigating noise source variations and that velocity change measurements should not be influenced by noise source variations. As shown by the evolution of the SNR of the correlations, with respect to different frequency bands (fig. S1), the period band of 3 to 10 s is the most energetic, with the SNR decreasing at longer and shorter periods. Therefore, we restrained our analysis to the period band of 3 to 10 s. The SNR was computed as the ratio between the maximum amplitude of the correlation in the 2- to 4-km/s direct wave arrival window and the root mean square of the coda slower than 2 km/s.

### *dv*/*v* measurement tests

The *dv*/*v* measurements were performed with both the doublet (*50*) and stretching (*14*, *25*) methods over the same window in the coda part of the correlations. This window has a *winlength* length in seconds, starting at a time *tminpercent* times the time of the maximum amplitude of the correlation, which is the direct surface wave arrival *t*_0_. Tests show that performing the *dv*/*v* measurement on both sides of the correlation simultaneously resulted in nonstable estimates of *dv*/*v* because the noise source distribution is not homogeneous. In the following, we only discuss the difference in measuring *dv*/*v* on the most energetic side of the correlation or on the symmetrized correlation (that is, after stacking the two sides). The reference correlation is the average of all daily correlations over the study period. The correlation for a specific day that we use for *dv*/*v* measurement is the average correlation of that day with the *daystack*-1 previous daily correlations. The influence of the parameters in italic font is discussed in the following.

For the doublet measurements, we followed the procedure of Clarke *et al*. (*50*), implemented in the MSNoise package (*46*). Inside the large *winlength* second-long window, we used small sliding windows with a length 10 times the central period of the signal. The small windows overlapped by 95%. For each small window, the cross-spectrum between the current and the reference correlation was computed. From this cross-spectrum, the coherence and the phase between the two signals as a function of the frequency were extracted. A weighted linear regression (weighted by the coherency) was performed on the phase in the frequency band 0.1 to 0.3 Hz to extract the phase delay between the reference and current correlation, as well as an error estimate in the slope. Thus, for each small sliding window, we obtained three values: a time delay (*tdelay*, in seconds), an error for the time delay (*errtdelay*, in seconds), and the average coherency between the two signals (*coh*). Then these measurements were used in a second step to evaluate the relative velocity variation *dv*/*v* = −*dt*/*t* between the reference and the current correlation. A weighted linear regression on the time delays with respect to the central time of the windows was used to calculate the final *dv*/*v* value and its uncertainty for a specific frequency band. Only the time delays *tdelay* < 0.5 s with errors *errtdelay* < 0.5 s and coherency *coh* > 0.75 were used in the final linear regression to estimate *dv*/*v*.

The stretching technique is based on the assumption that, if a small velocity change occurs homogeneously in the medium, then the current correlation will simply be a stretched or compressed version of the reference correlation. The stretching coefficient is therefore the relative velocity variation *dv*/*v*. Before the stretching measurement, the reference and current correlations were filtered in the frequency band 0.1 to 0.3 Hz. The measurement was performed using a grid search on the stretching coefficients. We sampled 100 stretching coefficients linearly spaced between −2 and 2%. For each coefficient, the time axis of the current correlation was stretched, and then the current correlation was interpolated onto this new time axis. The correlation coefficient between the window of the stretched current correlation and the reference correlation was then computed and stored. The best *dv*/*v* measurement was chosen as the stretching coefficient that maximized the correlation coefficient between the current stretched and reference correlations. To refine the estimation of *dv*/*v*, we used the maximum correlation coefficient and its nearest left and right neighbors. We performed a quadratic interpolation of these three points and took the stretching coefficient corresponding to the maximum of the interpolated curve. The error estimate was obtained from the expression derived by Weaver *et al*. (*51*). The error is related to the maximum correlation coefficient, the size and the position of the window in the coda, the frequency bandwidth, and the inverse of the central frequency of the signal.

Figure S6 shows the influence of the number of days used in the averaging to compute the current correlation. This sliding-window average is necessary to increase the coherence between the reference and the current correlation and to stabilize the *dv*/*v* measurement. Below 30 days, the results are not stable enough, and above 90 days, they are too smooth and lack structure to interpret (*52*).

The correlations are strongly asymmetric because of a nonhomogeneous distribution of noise sources around Greenland (*30*). It is possible that the less energetic side of the correlation did not converge toward Green’s function and is likely composed of random fluctuations. Therefore, we tested two measurement schemes: performing the measurements on (i) the most energetic side of the correlation and (ii) the symmetric part of the correlation (average of the causal and acausal parts). In our case, and as already observed by Witek *et al*. (*53*), averaging both sides of the correlations led to a decrease of coherency and a degradation of the signal. Even when the results were similar in both cases, we observed that the measurements from the symmetric part have larger uncertainties (fig. S7). Moreover, the difference between the doublet and stretching measurements was more pronounced in the symmetric case. Consequently, we chose to interpret only the measurements from the most energetic side, and on average, the *dv*/*v* measurements from the most energetic side appeared smoother and produced smaller errors. Next, we discuss the choice of the analysis window.

The analysis window is chosen in the coda part of the correlation, after the ballistic arrival, because coda waves are more sensitive to small changes in the medium. For instance, for a typical surface wave traveling at 3 km/s and a typical path of 300 km, the waves arriving 200 s after the direct wave have traveled a distance three times longer than the direct wave and are therefore three times more sensitive to changes in the medium. Moreover, the coda waves are much less sensitive to changes in the noise source properties and distribution (*54*). The velocity variation measurements performed on the coda waves are therefore much more reliable than the measurements performed on the direct waves (*55*). There are also trade-offs between the size and position of the window in the coda and the quality of the measurement. A window in the early coda benefits from the high coherency of the signal but suffers from the difficulty in measuring very small time delays. Later in the coda, the time delays are larger, but the poor coherency makes the measurement less reliable. The size of the window is not of major importance for the doublet measurements because the most uncertain values are discarded before estimating *dv*/*v*. However, the size of the window matters for the stretching technique because it directly influences the uncertainty estimation (*51*). At the frequencies used in this study (between 0.1 and 0.3 Hz), the Greenland crust was weakly scattering, resulting in a short coda with rapidly decreasing coherency (red curve above the reference correlation in figs. S3 and S6). In general, we observed that, 500 s after the direct wave arrival time, the average coherency between the reference and current correlations reaches the background noise level. Thus, the size of the window should be limited and should not extend past 500 s.

We tested two different approaches for the choice of the analysis window: a constant-length window and a variable-length window based on local coherency. For the variable-length window, we chose to end the window when local coherency went below 0.5. Figure S8 shows that the constant-length window (here, 300 s and starting 30% later than the direct arrival) exhibited smaller uncertainties. In the following analysis, we chose a 300-s-long window and tested different starting times in the coda (0, 10, 30, 50, 100, 150, and 200% the time of the direct arrival). Results are shown in fig. S9 for the station pair ILULI-NUUG. We observed that the first-order information was consistent for most of the windows, except for the window encompassing the main arrival (0%) and the very late coda (200%). We also observed that the uncertainties increased with the time in the coda, which was expected as the coherency decreased rapidly. From this analysis, it appeared that the windows starting at 10, 30, or 50% of the main arrival were the best compromise between stability of the retrieved variations, uncertainty level, and position in the coda.

Finally, at first order, the results from doublet and stretching methods appeared to be similar for individual pairs of stations (figs. S6, S10, and S11). However, the doublet measurements presented larger uncertainties and the stretching measurements were smoother and more easy to interpret. Moreover, the stretching measurements, using a long window in the coda, were more sensitive to large-scale, nonlocal changes, the type of changes that we were interested in. We thus continued using only the stretching measurements.

### GRACE data processing

We localized GRACE CSR (Center for Space Research) RL05 time-variable gravity data to a region in southwest Greenland using spherical Slepian basis functions (*9*). This method projects the monthly spherical harmonic coefficients (degree and order 60) onto a sparse basis of seven Slepian functions created specifically for this region (Fig. 1, main text). The Slepian functions optimally maximize their energy within the region of interest. With this method, we estimated the local gravity field changes within the region and minimized influence from the area outside the region, increasing the local SNR. The data were then spatially integrated across the chosen region, and the time series was detrended and filtered in the period band from 4 to 17 months, to be used as input to the poroelastic and viscous forward models.

## Supplementary Material

http://advances.sciencemag.org/cgi/content/full/2/5/e1501538/DC1

## SUPPLEMENTARY MATERIALS

Supplementary material for this article is available at http://advances.sciencemag.org/cgi/content/full/2/5/e1501538/DC1 fig. S1. SNR of the reference correlations. fig. S2. Seismic noise spectrograms. fig. S3. Characteristics of the analysis window used to measure the velocity variations for each station pair. fig. S4. Viscoelastic modeling: Vertical distribution of stress due to the ice sheet load. fig. S5. Estimation of *z*_t_ and *m*/μ. fig. S6. Influence of the number of correlations stacked. fig. S7. Influence of the symmetrization of the correlation on the *dv*/*v* measurements (ILULI-SFJ). fig. S8. Influence of the analysis-window length on the *dv*/*v* uncertainties. fig. S9. Effect of the analysis-window start time for the pair ILULI-NUUG (60-day stack, 0.1- to 0.3-Hz band, 300-s window). fig. S10. Example of doublet measurements for NRS-IVI and comparison with the stretching method. fig. S11. Example of doublet measurements for ILULI-SFJ and comparison with the stretching method.
