# Supplementary material for: Monitoring southwest Greenland’s ice sheet melt with ambient seismic noise
Source: Sci Adv. 2016 May 6;2(5):e1501538. doi: 10.1126/sciadv.1501538 (PMC4928992; doi:10.1126/sciadv.1501538)
Supplement: http://advances.sciencemag.org/cgi/content/full/2/5/e1501538/DC1 [file supp_2_5_e1501538__index.html]

Science Advances | Science Advances

## Supplementary Materials

**This PDF file includes:**

- fig. S1. SNR of the reference correlations.
- fig. S2. Seismic noise spectrograms.
- fig. S3. Characteristics of the analysis window used to measure the velocity variations for each station pair.
- fig. S4. Viscoelastic modeling: Vertical distribution of stress due to the ice sheet load.
- fig. S5. Estimation of *zt* and *m/*μ.
- fig. S6. Influence of the number of correlations stacked.
- fig. S7. Influence of the symmetrization of the correlation on the *dv/v* measurements (ILULI-SFJ).
- fig. S8. Influence of the analysis-window length on the *dv/v* uncertainties.
- fig. S9. Effect of the analysis-window start time for the pair ILULI-NUUG (60-day stack, 0.1- to 0.3-Hz band, 300-s window).
- fig. S10. Example of doublet measurements for NRS-IVI and comparison with the stretching method.
- fig. S11. Example of doublet measurements for ILULI-SFJ and comparison with the stretching method.

Download PDF

**Files in this Data Supplement:**

- Adobe PDF - 1501538\_SM.pdf
