# Supplementary material for: Monitoring southwest Greenland’s ice sheet melt with ambient seismic noise
Source: Sci Adv. 2016 May 6;2(5):e1501538. doi: 10.1126/sciadv.1501538 (PMC4928992; doi:10.1126/sciadv.1501538)
Supplement: http://advances.sciencemag.org/cgi/content/full/2/5/e1501538/DC1 [file 1501538_SM.pdf]

Science Advances

AAAS

advances.sciencemag.org/cgi/content/full/2/5/e1501538/DC1

Supplementary Materials for

Monitoring southwest Greenland's ice sheet melt with ambient seismic noise

Aurélien Mordret, T. Dylan Mikesel, Christopher Harig, Bradley P. Lipovsky, Germán A. Prieto

Published 6 May 2016, *Sci. Adv.* **2**, e1501538 (2016)
DOI: 10.1126/sciadv.1501538

The PDF file includes:

- fig. S1. SNR of the reference correlations.
- fig. S2. Seismic noise spectrograms.
- fig. S3. Characteristics of the analysis window used to measure the velocity variations for each station pair.
- fig. S4. Viscoelastic modeling: Vertical distribution of stress due to the ice sheet load.
- fig. S5. Estimation of z_t and m/μ.
- fig. S6. Influence of the number of correlations stacked.
- fig. S7. Influence of the symmetrization of the correlation on the *dv/v* measurements (ILULI-SFJ).
- fig. S8. Influence of the analysis-window length on the *dv/v* uncertainties.
- fig. S9. Effect of the analysis-window start time for the pair ILULI-NUUG (60-day stack, 0.1- to 0.3-Hz band, 300-s window).
- fig. S10. Example of doublet measurements for NRS-IVI and comparison with the stretching method.
- fig. S11. Example of doublet measurements for ILULI-SFJ and comparison with the stretching method.

Supplementary Materials

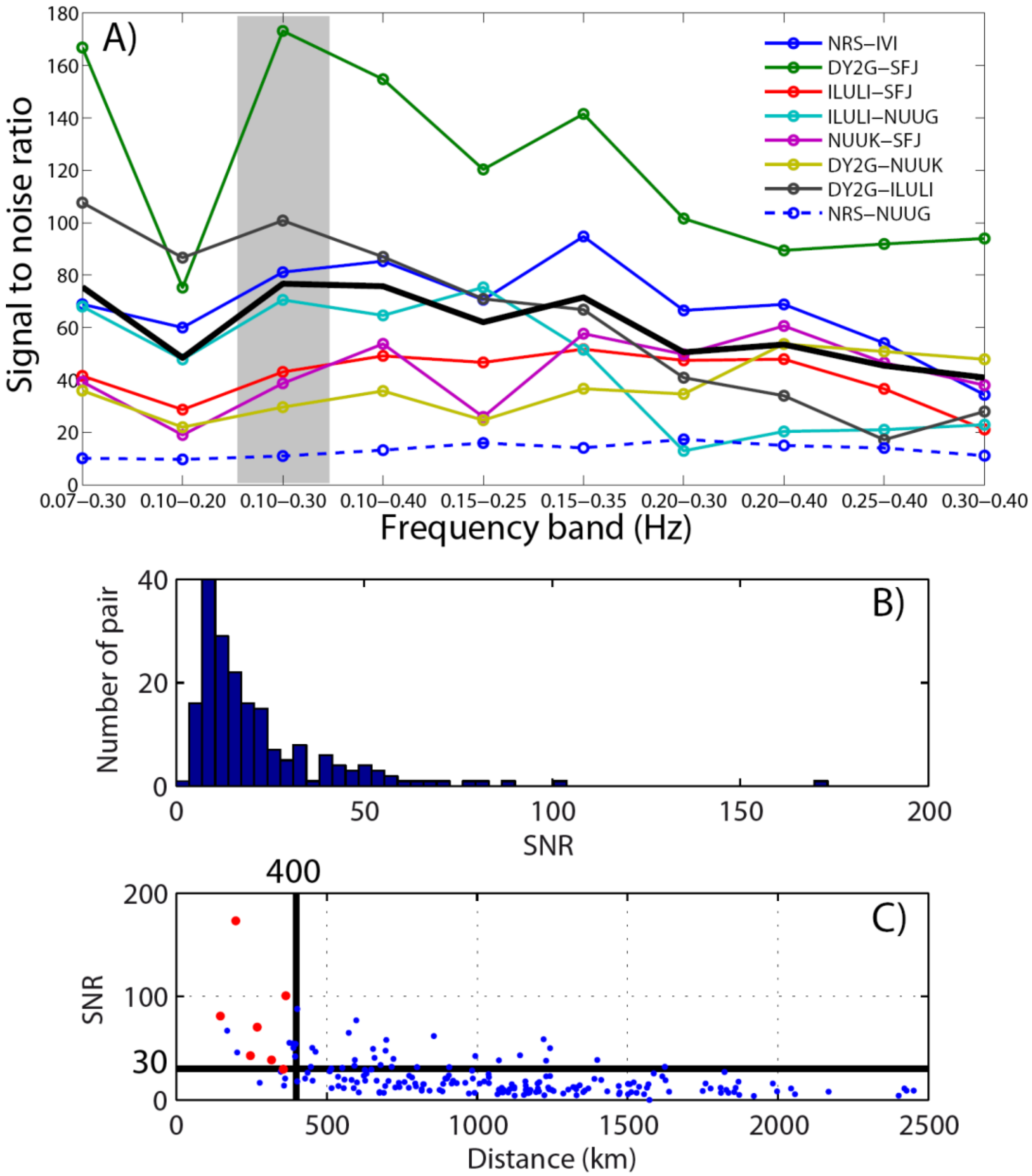

fig. S1. SNR of the reference correlations. (**A**) SNR for each station pair used in this study as a function of the frequency band. The thick black curve is the average SNR curve over the seven pairs of stations used in the study. The highest SNR is observed in the frequency band [0.1-0.3] Hz (highlighted by the gray shade). The dashed blue curve is the SNR curve for the pair NRS-NUUG, separated by 1200 km and not used in this study. The SNR is computed as the ratio between the maximum amplitude of the correlation in the 2-4 km/s direct wave arrival window and the root-mean-square of the coda slower than 2 km/s. (**B**) Distribution of the SNR (at [0.1-0.3] Hz) for every station pairs of the GLISN network: the mean value is ~20. (**C**) SNR (at [0.1-0.3] Hz) of every GLISN station pair as a function of the inter-station distance. The 7 pairs used in this study are in red. The horizontal and vertical black lines show the SNR and distance thresholds used to select the pairs.

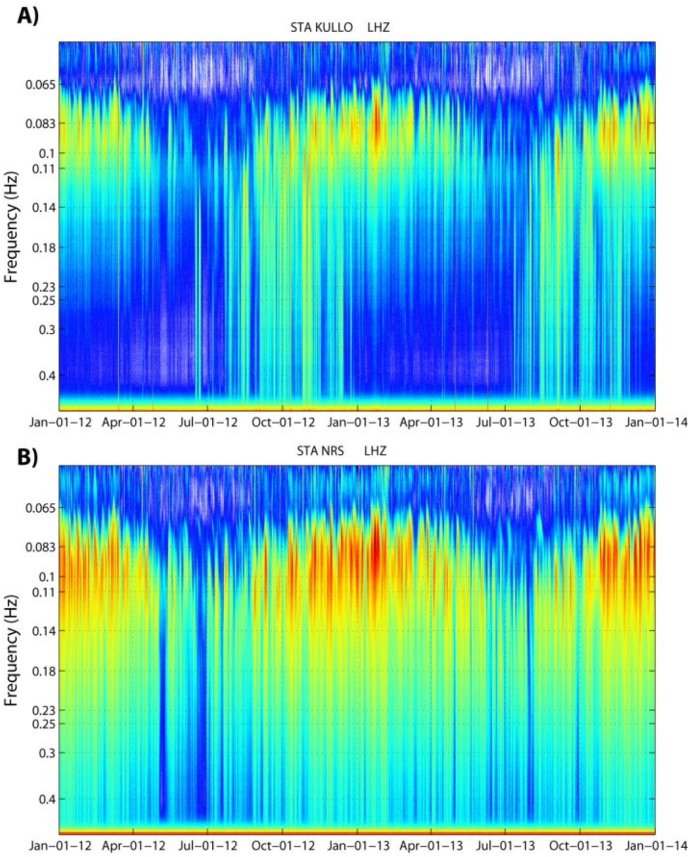

fig. S2. Seismic noise spectrograms. (**A**) Station KULLO. (**B**) Station NRS. The high frequency seismic noise disappears in winter and spring at station KULLO because of the presence of sea ice.

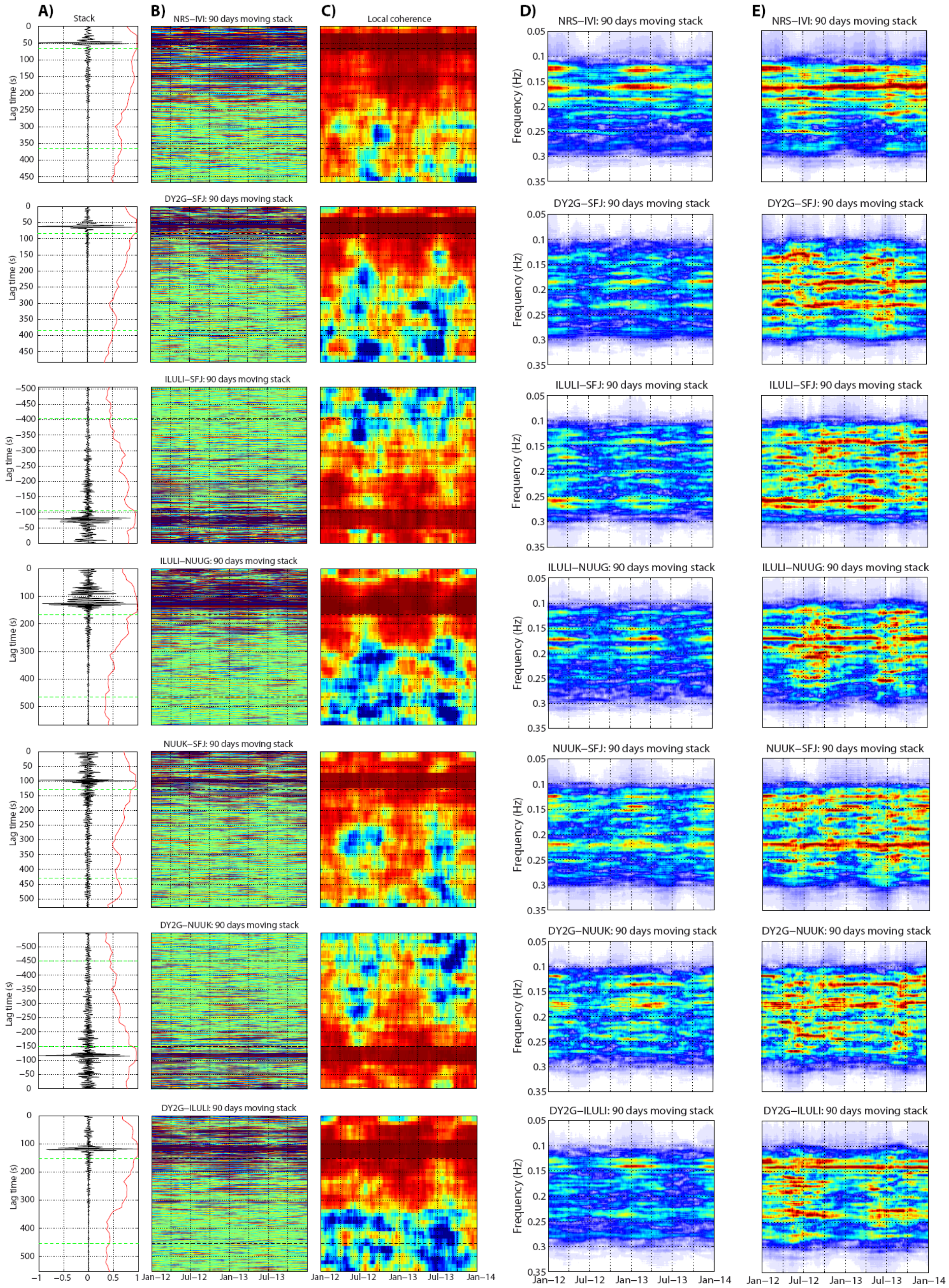
A)
Stack
B)
NRS–IVI: 90 days moving stack
C)
Local coherence
D)
NRS–IVI: 90 days moving stack
E)
NRS–IVI: 90 days moving stack
DY2G–SFJ: 90 days moving stack
ILULI–SFJ: 90 days moving stack
ILULI–NUUG: 90 days moving stack
NUUK–SFJ: 90 days moving stack
DY2G–NUUK: 90 days moving stack
DY2G–ILULI: 90 days moving stack
Lag time (s)
Frequency (Hz)
Jan–12
Jul–12
Jan–13
Jul–13
Jan–14

fig. S3. Characteristics of the analysis window used to measure the velocity variations for each station pair. (**A**) The reference correlation (the average of all daily correlations) and the window used to measure the velocity variations (shown in the panels A, B and C by the green and black dashed lines). (**B**) Daily correlations smoothed by a 90 days moving average. (**C**) Local coherence between the reference and each daily correlation measured in a moving window along the trace. The colors go from dark blue = 0 to dark red = 1. The red curve in the panel A) is the average of the local coherence. The data are filtered between 0.1 and 0.3 Hz. We observe a clear decrease in the coherence during summer months. This is reflected in the velocity variation measurements by the larger uncertainties in summer (see uncertainties in Figs. 1D and 2). (**D**) Amplitude spectra within the analysis window in the daily correlations filtered in [0.1-0.3] Hz band. (**E**) Same as (D) but amplitude normalized each day to highlight the continuity of the spectral peaks.

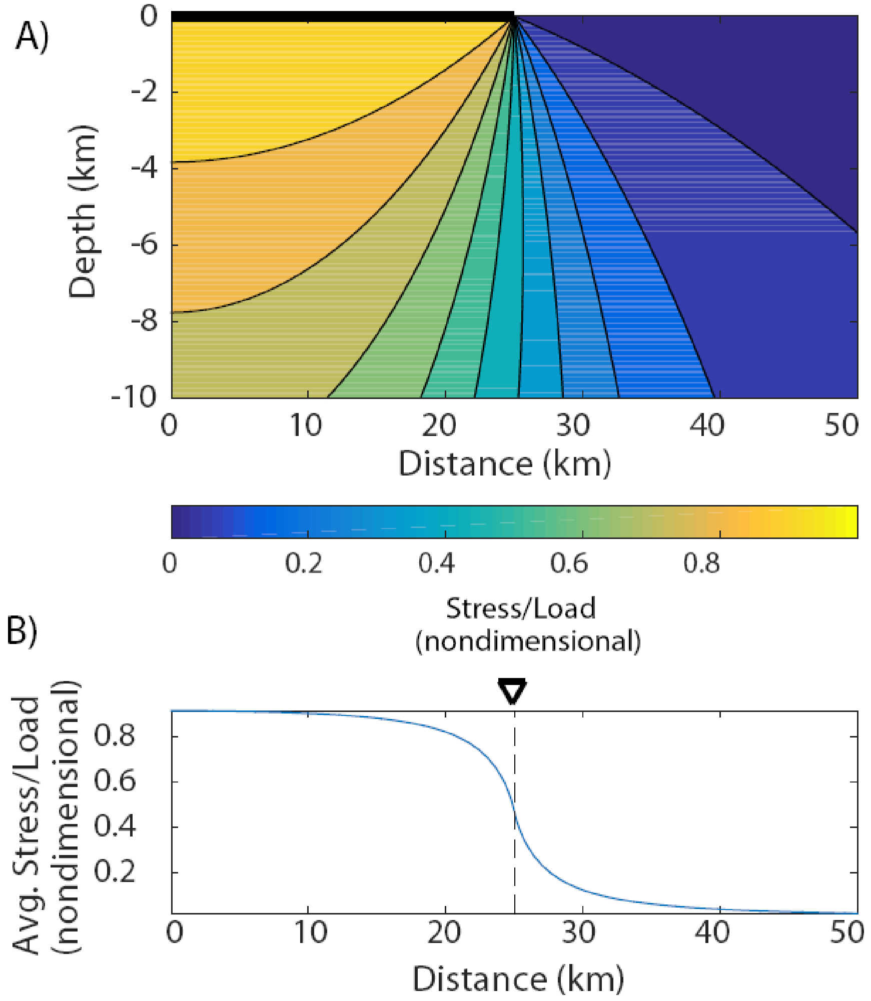

fig. S4. Viscoelastic modeling: Vertical distribution of stress due to the ice sheet load. (**A**) Vertical distribution of stress due to a load at the surface between 0 and 25 km (black line). (**B**) Stress averaged from 10 km depth to the surface. The inverted triangle shows the location of the seismic stations.

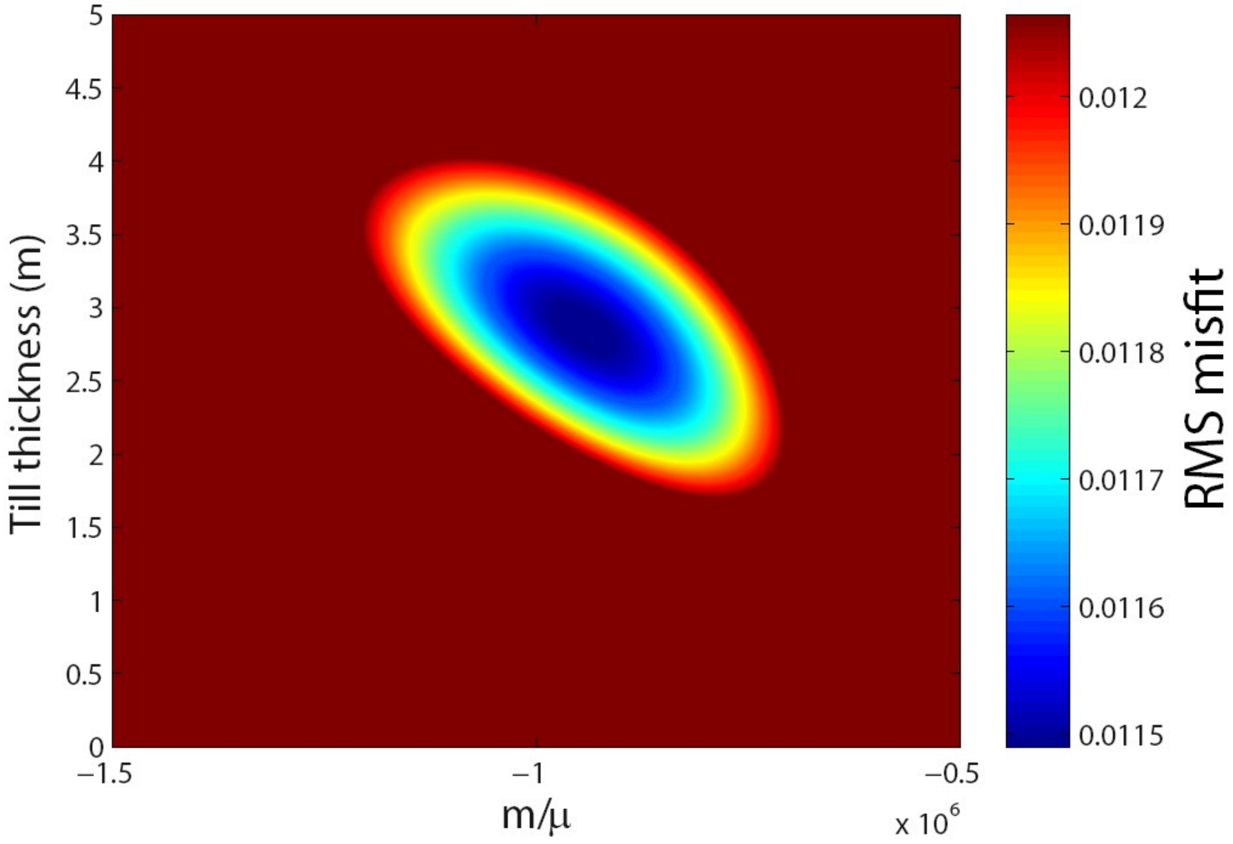

fig. S5. Estimation of z_t and m/μ. Misfit surface built from a grid search over the till thickness zt and the ratio of the Murnaghan constant and the shear modulus m/μ. Misfit is computed from the fit of the predicted *dv/v* and the measured *dv/v*. The best-fit values are z_t = 2.85 m and m/μ = -94.4·10^4.

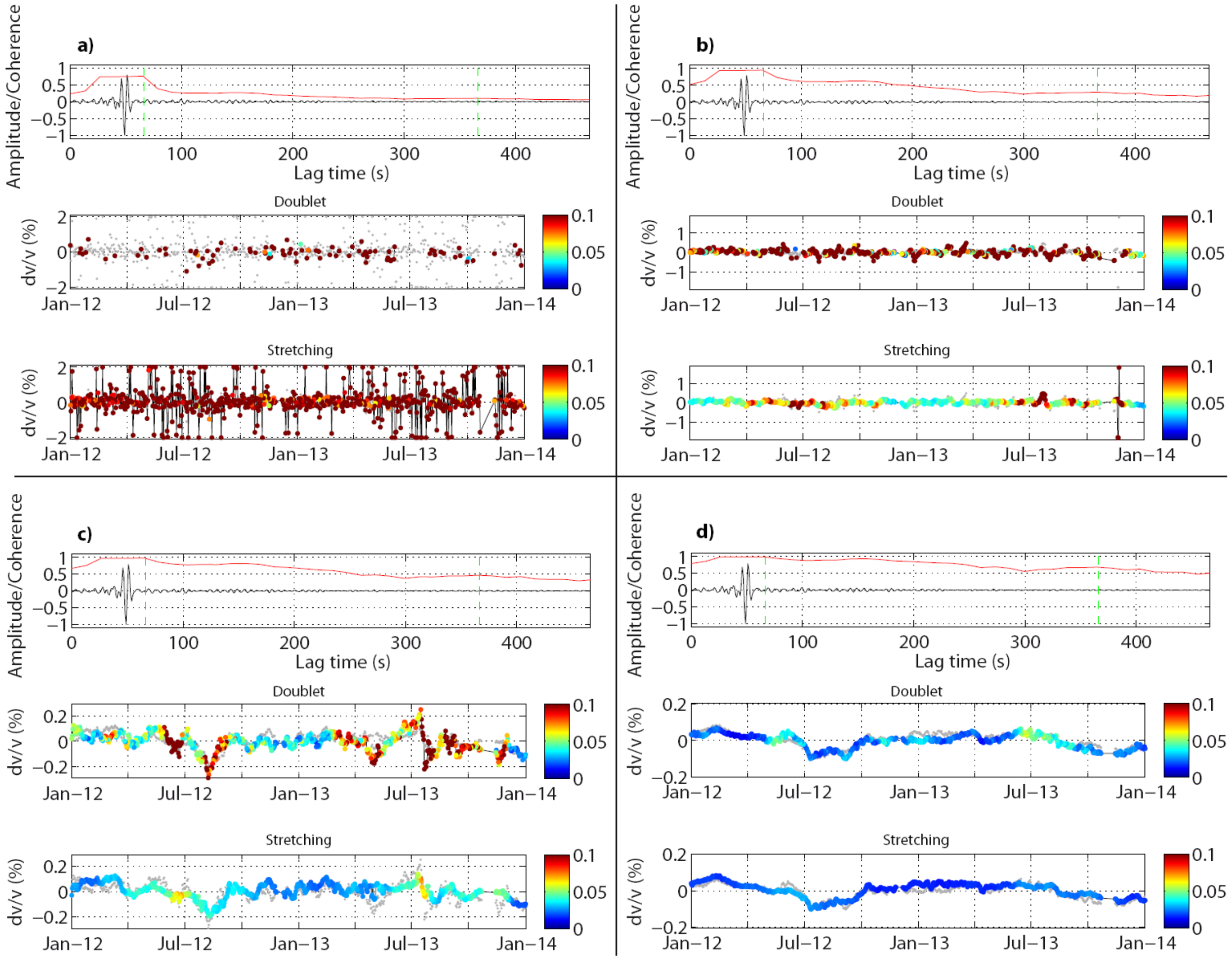

fig. S6. Influence of the number of correlations stacked. (**A**) 1-day stack. (**B**) 10-day stack. (**C**) 30-day stack. (**D**) 90-day stack. In each frame, the top row is the analysed correlation (NRSIVI) filtered between 0.1-0.3 Hz. The green lines show the analysis window in the coda used for the measurement, the red line is the average coherency between the reference correlation and all daily correlations (after the stack). The middle row shows the result for the doublet measurement in color and the result for the stretching measurements in gray. The bottom row shows the results from the stretching technique, with the doublet measurements in gray for comparison. The colorbar indicates the uncertainty.

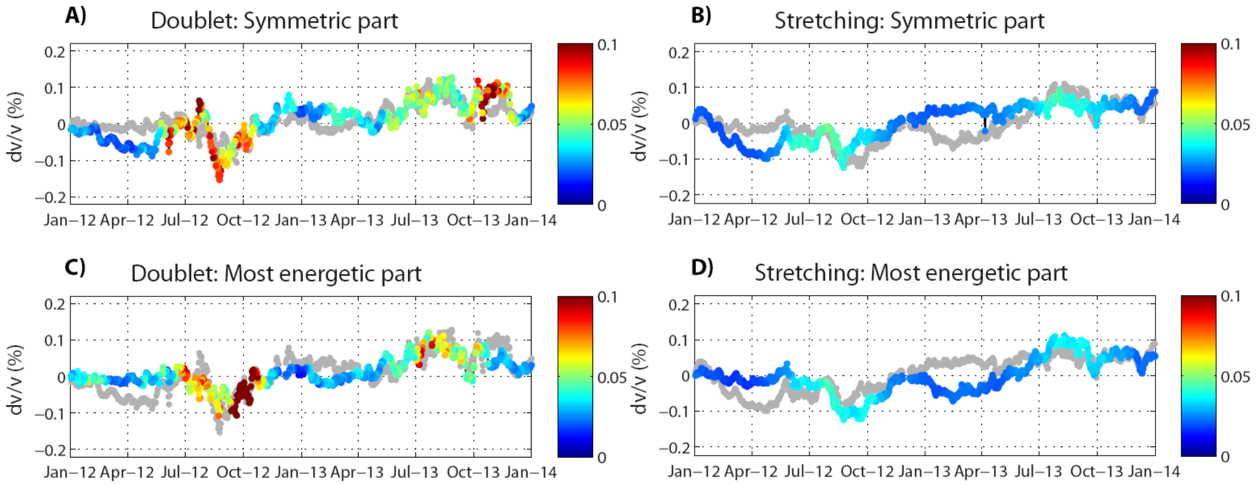

fig. S7. Influence of the symmetrization of the correlation on the *dv/v* measurements (ILULI-SFJ). (**A**) Measurement on the symmetric part with the doublet technique, 90 days stack 0.1-0.3 Hz. The measurements on the most energetic part are shown in gray. (**B**) Measurement on the symmetric part with the stretching technique, 90 days stack 0.1-0.3 Hz. The measurements on the most energetic part are shown in gray. (**C**) Measurement on the most energetic part with the doublet technique, 90 days stack 0.1-0.3 Hz. The measurements on the symmetric part are shown in gray. (**D**) Measurement on the most energetic part with the stretching technique, 90 days stack 0.1-0.3 Hz. The measurements on the symmetric part are shown in gray.

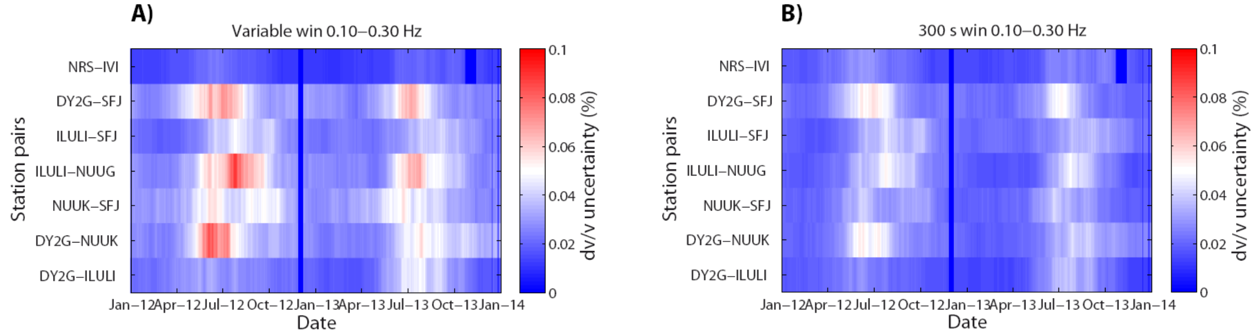

fig. S8. Influence of the analysis-window length on the *dv/v* uncertainties. (**A**) *dv/v* uncertainties obtained from a variable-length window using the stretching method, 90-day stack, 0.1-0.3 Hz band for all pairs. (**B**) *dv/v* uncertainties obtained with a constant length window for the stretching measurements, 90-day stack, 0.1-0.3 Hz, 300 s window for all pairs.

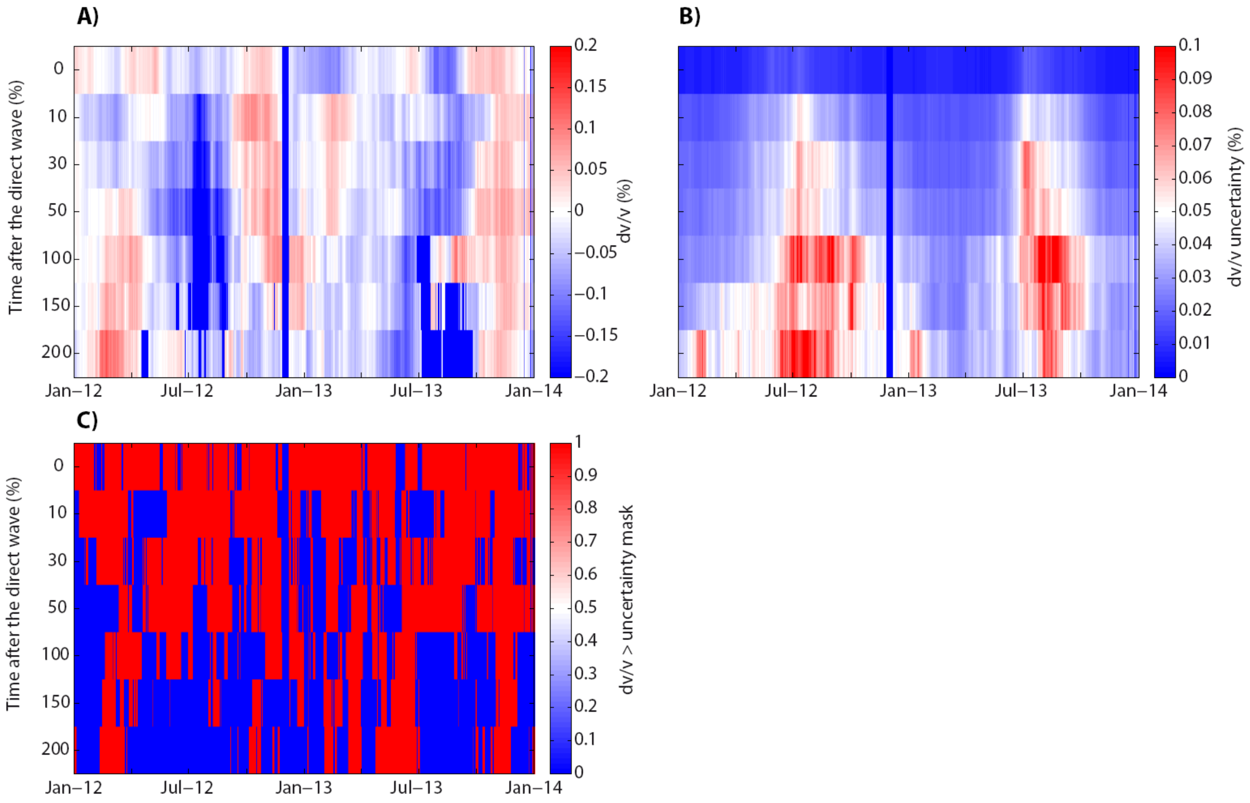

fig. S9. Effect of the analysis-window start time for pair ILULI-NUUG (60-day stack, 0.1- to 0.3-Hz band, 300-s window. (**A**) *dv/v* measurements for each tested start time. (**B**) *dv/v* uncertainties. (**C**) Mask showing where the *dv/v* measurements are larger than their uncertainties (in red).

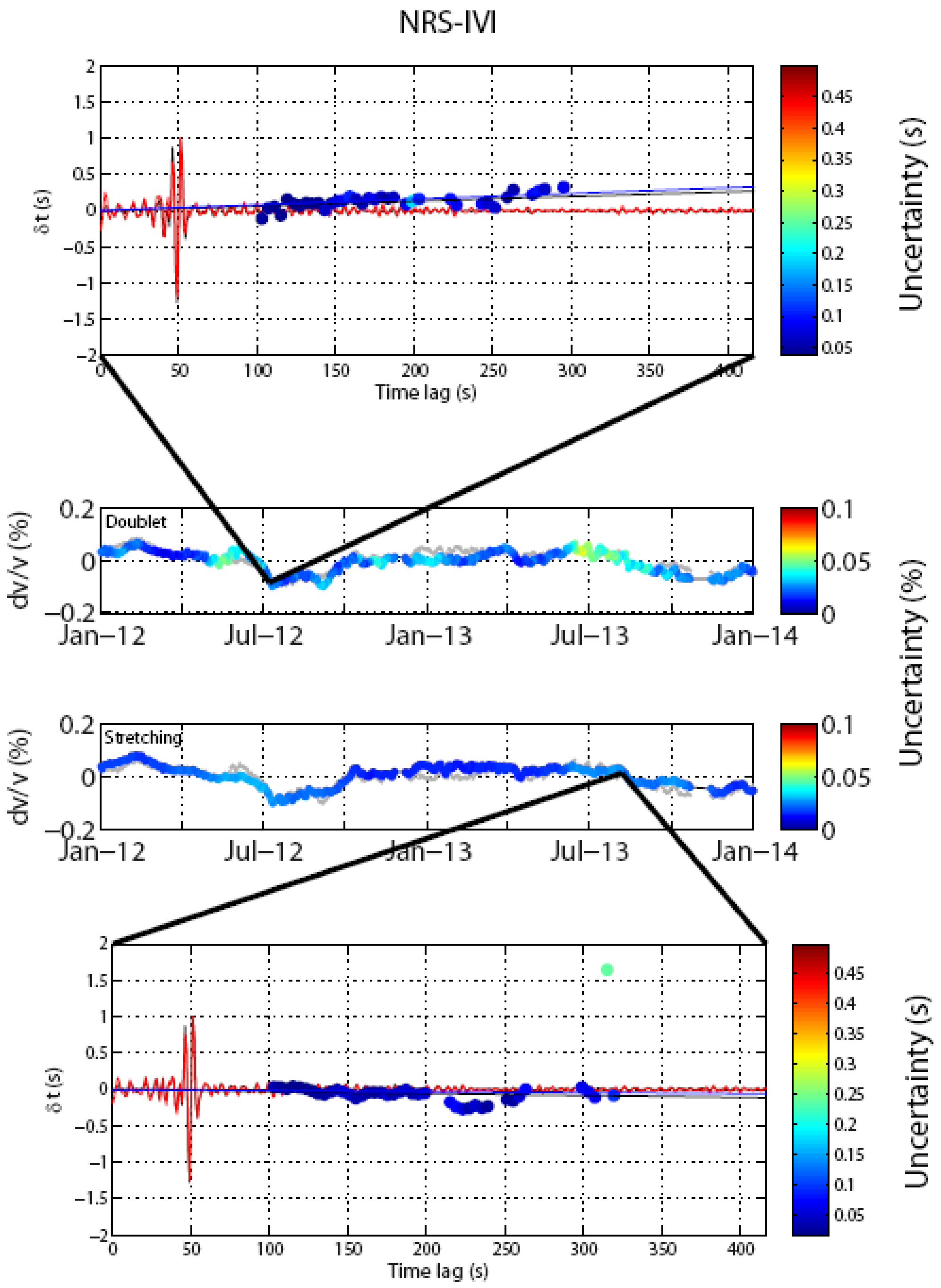

fig. S10. Example of doublet measurements for NRS-IVI and comparison with the stretching method. In the top and bottom panels, the current correlation is in red, the reference one, in black. The black straight line shows the linear regression of the δ*t* measurements, the blue straight line shows the δ*t*/*t* inferred from the stretching method. In the central panels, the gray dots are the measurements for the other method.

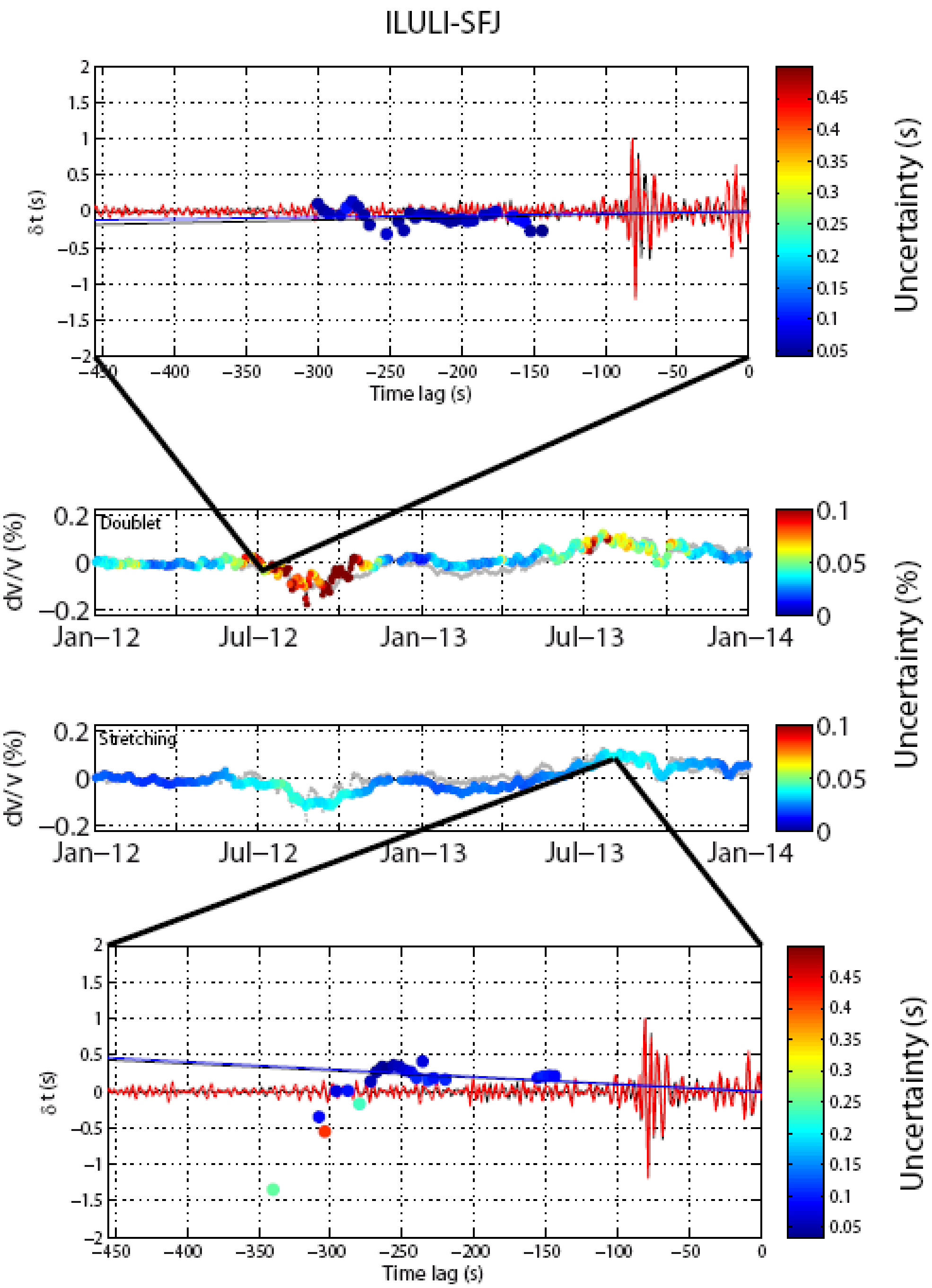

fig. S11. Example of doublet measurements for ILULI-SFJ and comparison with the stretching method. In the top and bottom panels, the current correlation is in red, the reference one, in black. The black straight line shows the linear regression of the δ*t* measurements, the blue straight line shows the δ*t*/*t* inferred from the stretching method. In the central panels, the gray dots are the measurements for the other method.
